# Evolving wastewater infrastructure paradigm to enhance harmony with nature

**DOI:** 10.1126/sciadv.aaq0210

**Published:** 2018-08-01

**Authors:** Xu Wang, Glen Daigger, Duu-Jong Lee, Junxin Liu, Nan-Qi Ren, Jiuhui Qu, Gang Liu, David Butler

**Affiliations:** 1 Research Center for Eco-Environmental Sciences, Chinese Academy of Sciences, Beijing 100085, China.; 2 Centre for Water Systems, College of Engineering, Mathematics and Physical Sciences, University of Exeter, Exeter EX4 4QF, UK.; 3 State Key Joint Laboratory of Environmental Simulation and Pollution Control, Research Center for Eco-Environmental Sciences, Chinese Academy of Sciences, Beijing 100085, China.; 4 Department of Civil and Environmental Engineering, University of Michigan, Ann Arbor, MI 48109, USA.; 5 Department of Chemical Engineering, National Taiwan University of Science and Technology, Taipei 10607, Taiwan.; 6 Department of Chemical Engineering, National Taiwan University, Taipei 10617, Taiwan.; 7 State Key Laboratory of Urban Water Resource and Environment, School of Environment, Harbin Institute of Technology, Harbin 150090, China.; 8 School of Environment, Tsinghua University, Beijing 100084, China.; 9 Sanitary Engineering, Department of Water Management, Faculty of Civil Engineering and Geosciences, Delft University of Technology, 2600 GA, Delft, Netherlands.

## Abstract

Restoring and improving harmony between human activities and nature are essential to human well-being and survival. The role of wastewater infrastructure is evolving toward resource recovery to address this challenge. Yet, existing design approaches for wastewater systems focus merely on technological aspects of these systems. If system design could take advantage of natural ecological processes, it could ensure infrastructure development within ecological constraints and maximize other benefits. To test this hypothesis, we illustrate a data-driven, systems-level approach that couples natural ecosystems and the services they deliver to explore how sustainability principles could be embedded into the life phases of wastewater systems. We show that our design could produce outcomes vastly superior to those of conventional paradigms that focus on technologies alone, by enabling high-level recovery of both energy and materials and providing substantial benefits to offset a host of unintended environmental effects. This integrative study advances our understanding and suggests approaches for regaining a balance between satisfying human demands and maintaining ecosystems.

## Teaser

This study advances our understanding of how to regain a balance between satisfying human demands and maintaining ecosystems.

## INTRODUCTION

Satisfying the ever-growing demands of humans while maintaining ecosystems is a long-standing challenge (*1*). Upgrading urban wastewater infrastructure is a case in point, as nearly 70% of the world population is expected to live in cities by 2050 (*2*) and, as cities continue to grow, the pressure on and unwanted effects of expanding wastewater service systems will increase significantly. Since the early 20th century, wastewater treatment has been implemented, improved, and subsequently optimized to ensure the safety of the aquatic systems and to minimize risks to human health (*3*). However, increasingly over the past decades, concerns have been raised over the unintended effects of historical approaches to wastewater service facilities. Natural resources, particularly fossil fuels, are consumed in the process of removing waterborne pollutants, and associated greenhouse gases (GHGs) are emitted. In the United States alone, nearly 3.4% of the generated electricity (15 GW) is used by wastewater treatment plants (WWTPs), representing the third largest consumer of electricity in that country (*4*). In a typical U.S. city, wastewater treatment can account for up to 24% of total energy usage by public utilities (*5*). Moreover, in the United States, CO_2_ emission of 0.6 gigatonnes (Gt) year^−1^ can be attributed to degradation of sewage organic matter over the period of 2010–2015. This amount is equivalent to ~1.5% of global emission and is projected to reach 1.0 Gt year^−1^ by 2050 (*6*, *7*).

Yet, in an evolving socioeconomic environment, the same waterborne and airborne contaminants could be considered valuable recycling resources. For example, organics could be used to produce sufficient energy to operate a WWTP (*8*). Roughly 3 million metric tons year^−1^ of phosphorus is lost as human waste, while ~20% of the global demand for phosphate can be satisfied by recovering phosphorus from this waste (*9*). Further, N_2_O is a common energy source in numerous applications, including automobile-related industries, where CO_2_ can be captured and synthesized for biomass production.

Wastewater resource management has attracted more attention and is included in a number of the United Nations (UN) Sustainable Development Goals (SDGs) (*10*). Despite ample opportunities, the transition of wastewater systems from a sole emphasis on pollutant removal to a focus on resource recovery is not easy to realize. This is partly because emerging concepts and methods are components of a complex integrated system intended to deliver broader benefits, including water reuse, nutrient recycling, and energy production, among others (*11*), while existing infrastructure paradigms have not been designed with these multiple purposes in mind. Moreover, wastewater service systems often function in isolation, relying only on technology to resolve problems and failing to address those factors beyond the traditional scope of engineering.

Currently, the enormous rise in urbanization and economic activity has compelled urban areas to increase their wastewater services. Since many wastewater infrastructure elements have service lives of 50 to 100 years, or even longer, the decisions made today have long-lasting implications and, consequently, must be based on future rather than current or past scenarios. To realize the potential for enhanced sustainability (*12*), the industry needs a fundamental change in its approach to and assumptions on managing wastewater resources, including creation of much-needed new-build wastewater systems (*13*). Accordingly, we illustrate a refined approach to integrate multiple options to reuse pollutants from used water as resources (referred to as REPURE infrastructure), with the following main features: (i) repurposing waterborne material (organic matter, nitrogen, and phosphorus, among other substances) to enable pollution control, resource capture, and end use of the harvested products; (ii) applying a sustainability philosophy to replace the traditional engineering method that focuses only on the technical aspect of system design; (iii) taking ecosystems into account to leverage the capabilities of the natural systems at the systems level.

**Fig. 1:**
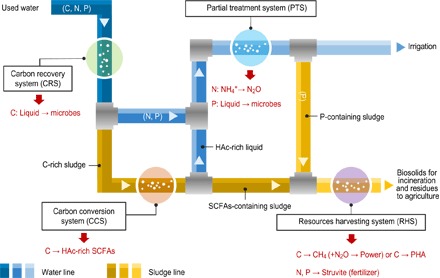
Overview of inputs, internal flows, and outputs of the REPURE approach. Most of the influent carbon substrates (C) are concentrated in the carbon recovery system (CRS), whereas the resulting C-rich biomass is fermented partly to acetic acid (HAc)–dominant short-chain fatty acids (SCFAs) in the subsequent carbon conversion system (CCS). The HAc-rich liquid serves as a promising carbon source in the partial treatment system (PTS) for nutrient removal. The resulting sludge from CCS and PTS is transformed to various products in the resources harvesting system (RHS). Most of the sludge C is converted to CH_4_, whereas the remainder is used for polyhydroxyalkanoate (PHA) synthesis. The wastewater N is converted to N_2_O, which is used for combustion with CH_4_ for power generation. The sludge P can be recovered as struvite.

To test the viability of this approach, we applied the REPURE concept to repurpose carbon (C), nitrogen (N), and phosphorus (P) in wastewater to attain higher resource efficiency (Fig. 1), building upon conventional and emerging concepts and methods in wastewater resource recovery. Next, we applied a rigorous dynamic process modeling (DPM) method to build the system characteristics of a sample REPURE scenario, and then tested the technical feasibility of the process configuration, taking into account the variations and uncertainties of multilevel parameters. Further, we used a substance flow analysis (SFA) tool to acquire aggregated data and to visualize the resource harvesting patterns and losses from the entire system. Finally, we used a probabilistic life cycle assessment (LCA) method to trace and assess the sustainability of the selected scenario and to outline an avenue for future wastewater service protocols in real-world contexts.

## MATERIALS AND METHODS

### Overall approach

**Fig. 2:**
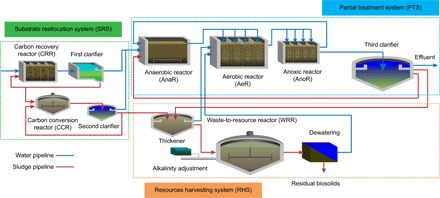
Schematic of a tailored process configuration for the REPURE approach. This configuration is constructed by three interlinked technological components (SRS, PTS, and RHS) to enable smooth operation and maintenance.

A tailored process configuration was established to examine and evaluate the REPURE approach. As illustrated in Fig. 2, the process configuration of the REPURE approach consisted of three main technological components—SRS, PTS, and RHS—to handle an example influent flow of 1 × 10^5^ m^3^ day^−1^, with a chemical oxygen demand (COD) of 400 mg liter^−1^, total nitrogen (TN) of 40 mg N liter^−1^, and total phosphorus (TP) of 7 mg P liter^−1^. Each of the three system components used a different set of reactors to provide the required functions. The key design factors for the system, along with the wastewater characteristics and environmental factors, among others, for the subsequent modeling and simulation, are provided in tables S1 to S3. Dynamic simulations were addressed in this work to satisfy a set of sample effluent quality requirements for a pollutant removal–oriented system (COD < 30 mg liter^−1^, TN < 15 mg N liter^−1^, NH_4_^+^-N < 5 mg N liter^−1^, and TP < 0.5 mg liter^−1^; these are stringent effluent limits in China) (*14*), although the effluent was considered a resource for recycling. Synergy between technological and ecological systems was included in the REPURE example, and two soil-mediated ecosystem services (carbon capture and nutrient retention) were characterized. Calculations are presented in tables S4 to S11.

### Inventory data and process models

The background inventory data on chemical, energy, and materials production are available in the literature (*4*, *7*, *12*, *15*) and the Ecoinvent libraries (*16*). The inventory data for the system elements, including system operation, were computed using model-based simulations. The DPM software BioWin version 5.0 (EnviroSim Associates Ltd.) was used to construct and simulate the physical, chemical, and biological processes involved in the selected configuration. The model factors were fixed initially, and a set of real-life dynamic influent data for a megacity in China was incorporated for the simulation (relevant statistic factors are provided in table S1). Considering the seasonal variations and slow dynamics of anaerobic processes, the evaluation period was extended to nearly 600 days (*17*). The details on determining the embodied and harvested energy and the GHG emissions, among others, are presented in the Supplementary Materials.

### Static SFA

A static SFA produces a systems-level overview of interlinked process and substance flows to design and assess the management options (*18*). Here, we quantified the C, N, and P fluxes by modeling the partitioning factors to water, air, and sludge for each process step in the water, sludge, and product pathways by using and aggregating input and output data derived from the process models. The SFA results were presented as a Sankey diagram prepared with the graphical software program Adobe Illustrator CS6 (Adobe Systems). Specifically, the width of each horizontal line on the figure was proportional to the flow of the substance. The mean values for the 600-day simulations were used to construct the diagram.

### LCA metrics

Twelve main LCA metrics were traced and assessed using the Hierarchist ReCiPe (H) midpoint method version 1.12, which is based on common policy principles, including the time frame (*19*). Specifically, midpoint methods relate the inventory results directly to environmental impacts, such as the climate change potential (*20*). As most of the recovered products were diverted to land use, terrestrial ecotoxicity was an interesting effect category to consider. As the ReCiPe method complies with all the critical aspects of human toxicity and includes terrestrial ecotoxicity, we selected it for use in our study. Slight modifications were made to fit the method closely with the goal of this work. Both climate change and ozone depletion included a characterization factor for N_2_O (298 kg CO_2_-eq kg^−1^ and 0.018 kg CFC11-eq kg^−1^, respectively), as N_2_O reportedly affected wastewater treatment and management alternatives in the wastewater industry (*21*). The adjusted ReCiPe approach was accessed subsequently in the LCA platform SimaPro (PRé Sustainability) to determine the LCA metrics.

### Hybrid DPM-SFA-LCA protocol

For integrative analyses, a hybrid DPM-SFA-LCA data integration approach was constructed by establishing interfaces to interconnect the three platforms using Python scripts from the literature (*22*). Python scripts integrated the results from BioWin over the simulation period to further aggregate the input and output data for SFA visualization in Adobe Illustrator C6. The integrated findings were transformed subsequently to a SimaPro-compatible input file for both foreground and background processes. The LCA metrics were calculated subsequently with SimaPro using the Ecoinvent databases. This final step measured the inventory results by adding the contribution of the background and foreground processes and subsequently determined the final LCA metrics using the modified ReCiPe method.

### Uncertainty accounting

It is essential for proper interpretation to model the attendant uncertainty with variations in several parameters, including inflow rate, influent characteristics, and environmental conditions. Here, we incorporated these variables using a probability-based method (*12*). Briefly, these direct inputs were fully integrated with the appropriate distributed uncertainty ranges for all the indirect inputs and emissions built for the processes. A Monte Carlo simulation analysis with 100,000 runs was also conducted in SimaPro to account for the effects of these parameter distributions on the overall LCA results. All the ranges and factor values are provided in the Supplementary Materials.

## RESULTS

### Repurposing water pollutants: What is the idea?

To enable the evolution of wastewater systems from a single focus on pollutant removal to the proposed recovery of resources, the leverage point is to redesign and realign the process configurations, aiming at repurposing the system inputs and diverting matter and energy flow from catabolism to anabolism. Building upon this theoretical basis and recovery technologies for wastewater resources, Fig. 1 shows the schematic flow of our REPURE design. Here, raw wastewater is fed initially into a CRS to concentrate the influent carbon substrates for further reallocation. This approach helps to address the major drawback of traditional activated sludge treatment processes, where wastewater organic matters are usually mineralized and their chemical energy potential [~1.9 kilowatt-hour (kWh) m^−3^] is typically consumed by energy-intensive aeration (0.3 to 0.7 kWh m^−3^) (*23*). Subsequently, C-rich biosolids are fermented into SCFAs in a CCS. This step provides triple benefits: (i) SCFAs are exceptionally promising substrates for nutrient-removing microbes (*24*), (ii) SCFAs serve as feedstocks for bioenergy and biochemical production (*25*), and (iii) wastewater systems fed with HAc-dominant SCFAs could achieve energy neutrality and enable a negative carbon footprint (*26*).

Subsequently, the combined stream from the CCS and CRS is treated to reduce the nutrient loads. Wastewater nitrogen is commonly removed by aerobic nitrification and subsequent anoxic denitrification, which requires energy from the mineralization of organic matters that could be used otherwise to produce energy carriers (for example, methane). Another challenge is the generation of N_2_O in these microbial processes (*27*). N_2_O is a critical GHG that is 310 times more powerful than CO_2_. Yet, N_2_O is also a common source of energy in numerous applications. Therefore, the PTS entails two essential steps: (i) conversion of NH_4_^+^ to N_2_O using ecological short circuits to reduce the oxygen supply and carbonaceous degradation (*28*) and (ii) conversion of N_2_O to N_2_, through which power can be generated by using N_2_O as an oxidant in methane combustion (*29*). Further, the fermentation liquid offers a feasible carbon source (HAc) to enable phosphorus accumulation in the waste-activated sludge by polyphosphate-accumulating organisms (PAOs) (*30*).

The treated effluent could be used for various nonpotable purposes, including agriculture (*31*), whereas the resulting biosolids will be processed in the RHS and converted into useful products. In particular, most organic matters in biosolids can be fermented into methane and combusted with the N_2_O for energy generation, while the PAOs in the RHS will take up the remaining SCFAs and store them intracellularly as PHA (*32*), which is a feasible substitute for petroleum-based plastics (*33*). Struvite (NH_4_MgPO_4_·6H_2_O) is harvested from the supernatant for use as a slow-release fertilizer (*34*). The digested biosolids are combusted to recover energy, and the residues are recycled to the land. The soil system receiving these products provides carbon capture and nutrient retention services (*35*, *36*).

### Can a REPURE example be formulated from concept to reality?

**Fig. 3:**
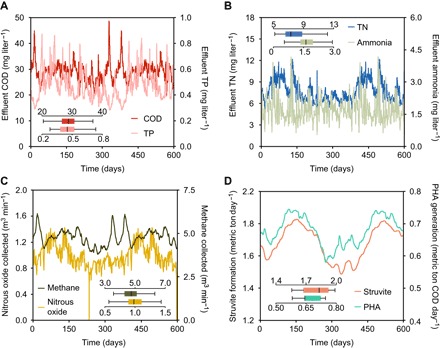
Long-term performance of the REPURE process configuration. Dynamic profiles of effluent COD and TP (**A**), effluent ammonia and TN (**B**), emitted nitrous oxide and methane (**C**), and formations of struvite and PHA (**D**) in the exemplified REPURE configuration. The small box plots in each chart depict the statistical information of the dynamic profiles, the central lines represent median values, the boxes represent the 25th to 75th percentiles, and the bars depict the 5th to 95th percentiles of the distributions resulting from the 600-day simulation.

To test the above-mentioned concept, we developed a tailored REPURE configuration (Fig. 2) and assessed it by applying a process modeling approach. The experimental differences were not considered in this study. The removal efficiencies of COD, TN, and TP were 92, 81, and 93%, respectively, during a 600-day evaluation period in the simulation model (table S12). In particular, the resulting water had 29 ± 4 mg COD liter^−1^, 7.8 ± 1.4 mg N liter^−1^, and 0.47 ± 0.11 mg P liter^−1^ (Fig. 3, A and B), with an effluent concentration of NH_4_^+^-N of 1.7 ± 0.5 mg N liter^−1^. This REPURE example complies with the accepted effluent limits while avoiding the common chemicals (iron and methanol) used in the traditional treatment processes (*37*).

In the SRS, the CRR firstly accumulated most of the organics in the influent waste stream to synthesize structural molecules and biomass via anabolism. The required metabolic energy is provided by the aerobic mineralization of the remaining organic matters and is relatively low. This is shown by the minor numbers of heterotrophs that used soluble biodegradable COD and exhibited low oxygen consumption (fig. S1). Next, the CCR converted the C-rich biosolids into HAc-rich substrates. In general, the removal of 1 mg of N and 1 mg of P consumes 6 to 8 mg and 7 to 10 mg of COD, respectively (*30*). Because of the high carbon content in the CCR, the influent COD into the PTS was obviously insufficient to satisfy biological nutrient removal. However, the reallocation of the carbon substrates and complementary addition of WRR fermentation liquid increased the HAc-based COD (table S13), which is one of the main reasons for the enhanced nutrient removal.

The expected shortening of the nitrification process was achieved in the PTS, as illustrated by the low NO_3_^−^ production (table S14) and corresponding weak metabolic activity of the nitrite-oxidizing bacteria (NOB) (table S15). Substantial N_2_O production is seen, with an average emission rate of 1.0 m^3^ min^−1^ (Fig. 3C, golden curve), equivalent to a harvesting rate of 2.6 metric tons N day^−1^. There are typically three main pathways involved in N_2_O formation: (i) NH_2_OH oxidation, (ii) nitrifier denitrification, and (iii) heterotrophic denitrification, mediated by the ammonia-oxidizing bacteria (AOB) and heterotrophs (fig. S2). The AOB-related N_2_O production pathways were predominant, as shown by the highest N_2_O production during autotrophic nitrification (4.4 mg N liter^−1^ hour^−1^; table S14). The metabolic data on AOB further demonstrated that NH_2_OH oxidation, rather than AOB denitrification, was the dominant pathway for generating N_2_O in the simulations (fig. S3). The fluxes of methane, struvite, and PHA are presented in Fig. 3 (C and D), indicating stable yield rates of 4.8 m^3^ min^−1^, 1.8 metric tons day^−1^, and 0.68 metric ton COD day^−1^, respectively. This finding indicates the feasibility of the HRS.

### SFA visualization: How many resources can be captured?

**Fig. 4:**
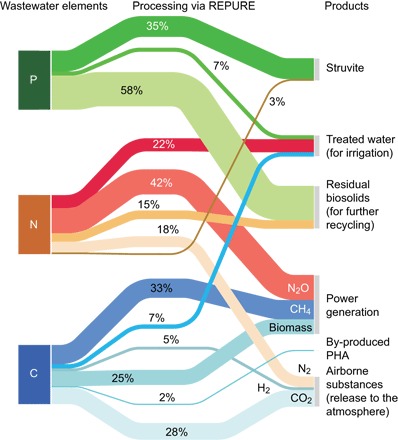
Sankey diagram tracing the intersystemic flows of C, N, and P from influent wastewater (from REPURE processing to the delivery of recovered products). The linewidth is proportional to the mass flux. The average values of the 600-day simulations were used to prepare this figure.

The result of the SFA (Fig. 4) indicates that most wastewater elements were consumed for energy production and were transformed into useful materials, with a relatively minor proportion of C and N lost to the atmosphere (28 and 18%, respectively, as biotic CO_2_ and N_2_). An examination of the fate of C (Fig. 4, blue series) indicates that 58% of the C load was converted into energy resources, with 33% converted to methane, and an additional fraction of 25% accumulated in the biosolids. Nearly 7% of the incoming C remained in the effluent, and a minor fraction of the C load was converted into PHA (2%). Regarding N (Fig. 4, orange-red series), 22, 15, and 3% remained in the effluent, biosolids, and struvite, respectively. This implies that 40% of the REPURE-based N fluxes can be recycled for land use. This is an advantage over the traditional biological nutrient removal systems, where most of the N_2_ is emitted to the atmosphere. Another major benefit is that the remaining 42% of the incoming N was converted into N_2_O as a powerful oxidant that could increase the energy harvesting efficiency by co-combustion with methane. Nearly 35% of the P load was used in struvite formation, whereas 58% of the incoming P accumulated in the biosolids for recycling in agriculture. A smaller proportion (7%) remained in the effluent (Fig. 4, green series).

**Fig. 5:**
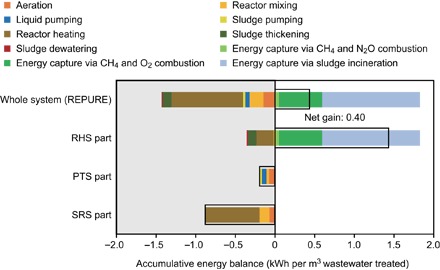
Energy balance and distribution in the REPURE process configuration. The left gray area in the chart indicates the energy required for system maintenance, whereas the right white area presents the energy produced from methane combustion and biosolid incineration. The black box presents the net energy gain relative to the system boundary considered, and the average values of the modeling results are shown.

This REPURE approach provides synergy, avoiding waste of biomass materials while enabling energy self-sufficiency. Emphasizing the accumulative energy balance for this configuration, Fig. 5 summarizes the energy embodied (left gray area) and exploited (right white area) across the subsystems. The embodied energy for system operation and maintenance was approximately 1.4 kWh m^−3^, whereas energy gained from methane exploitation and residue incineration completely satisfied the energy intake of the entire system, with a net power of 0.40 kWh m^−3^.

### Environmental sustainability: What are the effects?

**Fig. 6:**
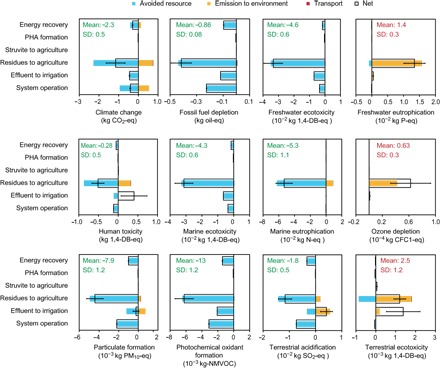
Net change in and processes contributing to 12 midpoint LCA effects, expressed per cubic meter of wastewater processed over 50 years of operation of the REPURE configuration. A negative value represents an environmental benefit, whereas positive values indicate an increase in the environmental burden. The relative size, or the apparent absence, of each color reflects the contribution of the process to each effect. The error bars present the best and worst cases of the pathway analyzed. The green or red text indicates statistically the net contribution of the system to each effect resulting from more than 100,000 Monte Carlo simulation runs, and the green means a net benefit for the environment.

Hitherto, we have addressed the technological feasibility and resource efficiency of this particular REPURE scenario. However, an assessment of the life cycle environmental effects is required. The probabilistic LCA results in Fig. 6 show that the REPURE approach provides environmental benefits by redirecting the used water resources. As regard the evaluated effects, the entire system contributes offsets to climate change (−2.3 kg of CO_2_-eq), fossil fuel depletion (−0.86 kg of oil-eq), freshwater ecotoxicity (−4.6 × 10^−2^ kg of 1,4-DB–eq), human toxicity (−0.28 kg of 1,4-DB–eq), marine ecotoxicity (−4.3 × 10^−2^ kg of 1,4-DB–eq), marine eutrophication (−5.3 × 10^−2^ kg of N-eq), particulate formation (−7.9 × 10^−3^ kg of PM_10_-eq), photochemical oxidant formation [−13 × 10^−3^ kg-NMVOC (nonmethane volatile organic compound)] and terrestrial acidification (−1.8 × 10^−2^ kg of SO_2_-eq), expressed per cubic meter of wastewater treated.

This REPURE case realized 100% energy self-sufficiency by diverting wastewater organics for energy generation. Furthermore, the contribution of the system to climate change and fossil fuel depletion was reduced, although CO_2_ was still released from the generation of bioenergy (fig. S4). Furthermore, diversion of the excess energy captured from the system to other urban sectors could assist in limiting the use of fossil fuel, thereby helping to reduce the attendant GHG emissions (0.10 kg oil-eq m^−3^ and 0.42 kg CO_2_-eq m^−3^). The exploitation of bioenergy plays an essential role in reducing the negative consequences associated typically with fossil fuel exploitation, such as particulate formation, photochemical oxidant formation, and terrestrial acidification.

Further, using the remaining biosolids for agriculture could help reduce the most negative effects by restricting the production and utilization of commercial fertilizers, except for ozone depletion, freshwater eutrophication, and terrestrial ecotoxicity. In particular, the gaseous emissions of CH_4_ and N_2_O from land use increase the ozone depletion potential. By applying the products to land use, the issue of nutrient discharge will be transferred from the aquatic ecosystems to the environment at the site of application. While 95% of P in biosolids could be absorbed by soils (see the Supplementary Materials), the remaining P is unavailable to the land and will run off, presenting a freshwater eutrophication potential of 1.6 × 10^−2^ kg P-eq m^−3^. In addition, recycling the biosolids for land use poses the risk of potential terrestrial ecotoxicity, owing to the metals in the biosolids rather than the organic pollutants, as most of the latter will be degraded during incineration. Considering the relatively lower yields of both PHA and struvite, it is not surprising that their effects are negligible.

This analysis assumed that the effluent was used as an alternative irrigation source. Hence, it mitigated the potentials for climate change and fossil fuel depletion. Further, diverting the effluent from the receiving water bodies to land use would benefit the aquatic ecosystems, considering its negligible eutrophication potential. In addition, the dissolved ammonia was released with the effluent irrigation, resulting in a net terrestrial acidification potential of 0.41 kg SO_2_-eq m^−3^. Moreover, effluent irrigation added metals and organic contaminants to soils, causing net potential terrestrial ecotoxicity (1.4 × 10^−3^ kg 1,4-DB–eq m^−3^) and human toxicity (0.40 kg 1,4-DB–eq m^−3^). Nevertheless, other uses for the reclaimed water could similarly reduce the application of traditional water resources and produce other net benefits.

## DISCUSSION

The 17 SDGs under *Agenda 2030* of the UN have mapped a coherent path and reached consensus on achieving global sustainability. Reusing wastewater has become an essential target across several SDGs, particularly in Clean Water and Sanitation (Goal 6). Given the time frame of less than 13 years, progress toward achieving the SDGs requires the effective conversion of evolving knowledge into practical solutions. Many countries have outlined a range of programs and actions to transform the existing wastewater treatment infrastructure into resource recovery facilities (*38*). Our findings indicated that a systems integration approach to develop complete systems allowed this ongoing revolution to produce significantly superior outcomes in a real-world context. This is in contrast to a more traditional approach based on the qualitative assumption that “more is better,” in which simply adding many alternative options is believed to lead to a sustainable wastewater treatment system. Combining various technological components into a complete system and assessing these systems systematically facilitate the development and selection of wastewater systems that provide superior net resource sustainability. Furthermore, these systems can reduce the negative consequences of and offset climate change, fossil fuel depletion, aquatic ecotoxicity, and additional broader effects. Although diverting reclaimed water and biosolids to productive uses can create benefits, it could also shift seemingly unrelated effects across systems and scales. To overcome these obstacles, an approach that is much more quantitatively rigorous and ecologically inclusive should be considered in the planning and design of wastewater infrastructure, from conception to configuration and analysis at the systems level. Such an ideal approach is presented by the current method illustrated through the REPURE example.

Global sustainability challenges are closely linked yet often considered and dealt with separately (*39*). The potential of wastewater resource infrastructure for effective coupling with natural ecosystems should be explored by considering both the emissions and the recovered products. A holistic methodology to study the coupling of technical and ecological systems is needed to advance our understanding and methods of creating truly sustainable wastewater management protocols. Here, we included the two most common ecosystem services (carbon capture and nutrient retention) provided by soils, as they were found to help reduce environmental effects during land use of the biosolids and reclaimed water. This approach could be promoted for many sites, as soil is a major component of the planet and exists in nearly every country. Although only two ecosystem services provided by soils were included at the systems level, using simple calculation parameters, the results showed that coupling technical systems and ecosystems provided the potential to pinpoint novel and mutually beneficial solutions that might not be discovered by a traditional technocentric approach. Nevertheless, expansion of this approach is not only possible but also necessary. For instance, the coupling of technologies and ecosystems should consider local and larger scales and include additional ecosystems, such as trees, to close more resource loops to manage wastewater treatment infrastructure in a sustainable manner. Advanced algorithms to describe, simulate, and predict ecosystem service benefits should also be integrated in future studies.

The expected products generated by the REPURE system mainly include energy carriers, biosolids, and reclaimed water. Exploitation of the renewable energy carriers (such as CH_4_) for power is the most common action, particularly as the harvested energy is used immediately onsite for plant operations. Further, biosolids from wastewater facilities have been commonly used worldwide for the recycling of organic matters and nutrients in agricultural fields, either directly via land spread or through composting. In addition, reclaimed water is increasingly applied for a variety of nonpotable purposes, including land irrigation, as assumed in this study. Another two REPURE products, struvite and biopolymer, are still in their infancy, with realization being hampered partly by economic or technical constraints. However, this study has generated a complete and generalized example of an approach to recover substantial amounts of wastewater energy and materials. It should be noted that our scenario can be upgraded according to actual needs and technology development. For example, in this simulation, approximately 28% of wastewater organics was converted to biotic CO_2_. Additional approaches, such as microalgal systems, could be integrated with the current scenario to enhance energy balances and substantially reduce onsite carbon emissions, as algae cultivation is able to capture CO_2_ and produce algae biofuels.

Here, the integrated analysis of the emerging approach relies on the hybrid models. Such a computer-based analysis conducted at an early stage of any substantial practice could help pinpoint promising avenues for wastewater resource recovery facilities. In addition, it can direct timely infrastructure investments that would be adequate for future scenarios. However, our models could be refined further once more data are made available. Here, the major source of uncertainty derived from the use of the ReCiPe model itself, which is the basis for the impact characterization conducted in the LCA analysis. Although the toxicity models include metals (*40*), many emerging contaminants are still excluded from current models. Recent advances in the ReCiPe model feature characterization factors for more organic contaminants, although this model still incorporated only 55% of the 110 organic contaminants in biosolids identified from the literature. In addition, nearly 30% of up to 300 organic pollutants were identified in graywater or treated water. Although many organic contaminants were still excluded, previous results suggested that, on the basis of the existing toxicity models, the inclusion of additional organic pollutants would probably not alter the human toxicity potential of any case study (*41*). Yet, the terrestrial and freshwater ecotoxicity potentials of biosolid use could be sensitive to the inclusion of other organic pollutants. Therefore, further research is required.

Although the REPURE approach has significant potential to sustain wastewater infrastructure transformation, subsequent studies are needed to verify this approach at a pilot scale. An ideal pilot REPURE facility must be fully integrated and should be able to assess the whole system while having sufficient flexibility to explore alternative configurations and to test options for improved system integration and recycling of the element streams (including C, N, and P). Such a pilot facility could be operated in a specific location, but its products can be used elsewhere across regions or even nations. Therefore, such a large coupled system, including both resource recovery and utilization, must be piloted initially with specific local conditions to facilitate easy testing. Our REPURE system produced renewable energy during wastewater handling and diverted the treated water from discharge to land use. We found that aquatic-eutrophic regions usually collocated with large energy consumers, such as food processing plants, pulp and paper plants, refineries, and agriculture. Siting near energy consumers an ideal location for the pilot REPURE facility, as it reuses the treated effluent and alleviates the local eutrophication pressure. Further, it also provides renewable energy for the energy-intensive industries located nearby to save more applied power, while the neighboring plants can help to address the end use of the materials produced from the REPURE facility.

In this analysis, we used broad site parameters with a wide range of variables in order to explore the applicability of our approach to a broad range of situations. Accordingly, it can be expected that the results presented, particularly those related significantly to the process performance and resource efficiency of the approach, would likely not be altered when the approach is implemented under different spatial conditions. Nevertheless, further study should concentrate on scenario analysis of this approach at a global scale, as potentially valuable insights could be gained from a thorough exploration, with spatial variations, of this new-build approach. For example, differences might be encountered in the LCA findings, especially those with close relevance to the assumptions of both carbon sequestration and nutrient retention. Such differences could arise because the capabilities of soils to provide these two ecosystem services could vary across regions. Notwithstanding the potential benefits, such an enterprise is data-intensive, making high demands on both the amount and quality of the underlying data (*42*). Further exploration of our approach at a global scale requires spatially explicit input data of extremely diverse types and from many different sources, including local climate, demographics, socioeconomic factors, water quality, soil characteristics, and system performance, among others (*43*). The increased availability of manifold data in Europe or North America allows the reliability and generality of our approach to be verified explicitly at a spatial scale. However, slow advances in analytics, sensing, monitoring, and computing and data management still exist in many places around the world (*44*, *45*), particularly in India and sub-Saharan Africa, making data acquisition rudimentary and tedious. Appropriate protocols are needed in these places to address data collection, use, and sharing, which would provide more extensive and more reliable data to facilitate infrastructure transformation in the global water sector.

Overall, large-scale application of the REPURE approach needs the following: (i) aggregation of more reliable data from diverse conditions, coupled models from wastewater engineering, LCA, and ecological modeling; (ii) advances in traditional disciplines, for example, the economic feasibility of totally new methods for water resource recovery should be analyzed carefully in comparison with those methods aimed to retrofit existing facilities, as conversion cost is a critical constraint of infrastructure transformation; and (iii) multidisciplinary collaboration and industry engagement. Many opportunities exist for theoretical and applied studies to develop sustainable wastewater management protocols. Our present study provides essential information to a broad multidisciplinary audience to build effective solutions to improve the harmony between humans and nature, with the goal of regaining the balance between satisfying human needs and protecting ecosystems.

## Supplementary Material

http://advances.sciencemag.org/cgi/content/full/4/8/eaaq0210/DC1

## SUPPLEMENTARY MATERIALS

Supplementary material for this article is available at http://advances.sciencemag.org/cgi/content/full/4/8/eaaq0210/DC1 Supplementary Text Fig. S1. Profiles of oxygen consumption and active microbes in CRR. Fig. S2. Simplified illustration of the three key N_2_O production pathways by AOB and heterotrophic denitrifiers. Fig. S3. Growth and decay rates of AOB in the PTS reactors. Fig. S4. Carbon footprint during operation of the REPURE process configuration. Table S1. Environmental parameters and main characteristics of influent for process design and modeling. Table S2. Design parameters for the developed technological configuration. Table S3. Construction inventory data for the REPURE process configuration. Table S4. Default assumptions for gaseous emissions and attendant variability for uncertainty analysis. Table S5. Heavy metal contaminants in biosolids. Table S6. Metal contaminants in struvite. Table S7. Heavy metal concentrations in treated effluent. Table S8. Organic contaminants in biosolids. Table S9. Organic contaminants in treated effluent. Table S10. Assumed availability of nutrients in recovered fertilizers as a fraction of commercial fertilizer availability. Table S11. Transport assumption and distances for recovered and commercial fertilizers. Table S12. Removal efficiencies of effluent COD, TN, and TP for the REPURE system. Table S13. Comparison of the average concentration of the major carbon substances in the outflow from CRR, CCR, and RHS with influent wastewater. Table S14. Average removal and production rates of different nitrogen species in the PTS reactors. Table S15. Metabolism of NOB in the three PTS reactors. References (*46*–*93*)
