# Supplementary material for: Evolving wastewater infrastructure paradigm to enhance harmony with nature
Source: Sci Adv. 2018 Aug 1;4(8):eaaq0210. doi: 10.1126/sciadv.aaq0210 (PMC6070318; doi:10.1126/sciadv.aaq0210)
Supplement: http://advances.sciencemag.org/cgi/content/full/4/8/eaaq0210/DC1 [file supp_4_8_eaaq0210__index.html]

Science Advances | Science Advances

## Supplementary Materials

**This PDF file includes:**

- Supplementary Text
- Fig. S1. Profiles of oxygen consumption and active microbes in CRR.
- Fig. S2. Simplified illustration of the three key N2O production pathways by AOB and heterotrophic denitrifiers.
- Fig. S3. Growth and decay rates of AOB in the PTS reactors.
- Fig. S4. Carbon footprint during operation of the REPURE process configuration.
- Table S1. Environmental parameters and main characteristics of influent for process design and modeling.
- Table S2. Design parameters for the developed technological configuration.
- Table S3. Construction inventory data for the REPURE process configuration.
- Table S4. Default assumptions for gaseous emissions and attendant variability for uncertainty analysis.
- Table S5. Heavy metal contaminants in biosolids.
- Table S6. Metal contaminants in struvite.
- Table S7. Heavy metal concentrations in treated effluent.
- Table S8. Organic contaminants in biosolids.
- Table S9. Organic contaminants in treated effluent.
- Table S10. Assumed availability of nutrients in recovered fertilizers as a fraction of commercial fertilizer availability.
- Table S11. Transport assumption and distances for recovered and commercial fertilizers.
- Table S12. Removal efficiencies of effluent COD, TN, and TP for the REPURE system.
- Table S13. Comparison of the average concentration of the major carbon substances in the outflow from CRR, CCR, and RHS with influent wastewater.
- Table S14. Average removal and production rates of different nitrogen species in the PTS reactors.
- Table S15. Metabolism of NOB in the three PTS reactors.
- References (*46*–*93*)

Download PDF

**Files in this Data Supplement:**

- Adobe PDF - aaq0210\_SM.pdf
