# Supplementary material for: Evolving wastewater infrastructure paradigm to enhance harmony with nature
Source: Sci Adv. 2018 Aug 1;4(8):eaaq0210. doi: 10.1126/sciadv.aaq0210 (PMC6070318; doi:10.1126/sciadv.aaq0210)
Supplement: http://advances.sciencemag.org/cgi/content/full/4/8/eaaq0210/DC1 [file aaq0210_SM.pdf]

Science Advances
AAAS

advances.sciencemag.org/cgi/content/full/4/8/eaaq0210/DC1

Supplementary Materials for

Evolving wastewater infrastructure paradigm to enhance harmony with nature

Xu Wang*, Glen Daigger, Duu-Jong Lee, Junxin Liu, Nan-Qi Ren, Jiuhui Qu, Gang Liu, David Butler

*Corresponding author. Email: xuwang@rcees.ac.cn; x.wang@exeter.ac.uk

Published 1 August 2018, *Sci. Adv.* **4**, eaaq0210 (2018)
DOI: 10.1126/sciadv.aaq0210

This PDF file includes:

Supplementary Text
Fig. S1. Profiles of oxygen consumption and active microbes in CRR.
Fig. S2. Simplified illustration of the three key N_2O production pathways by AOB and heterotrophic denitrifiers.
Fig. S3. Growth and decay rates of AOB in the PTS reactors.
Fig. S4. Carbon footprint during operation of the REPURE process configuration.
Table S1. Environmental parameters and main characteristics of influent for process design and modeling.
Table S2. Design parameters for the developed technological configuration.
Table S3. Construction inventory data for the REPURE process configuration.
Table S4. Default assumptions for gaseous emissions and attendant variability for uncertainty analysis.
Table S5. Heavy metal contaminants in biosolids.
Table S6. Metal contaminants in struvite.
Table S7. Heavy metal concentrations in treated effluent.
Table S8. Organic contaminants in biosolids.
Table S9. Organic contaminants in treated effluent.
Table S10. Assumed availability of nutrients in recovered fertilizers as a fraction of commercial fertilizer availability.
Table S11. Transport assumption and distances for recovered and commercial fertilizers.
Table S12. Removal efficiencies of effluent COD, TN, and TP for the REPURE system.
Table S13. Comparison of the average concentration of the major carbon substances in the outflow from CRR, CCR, and RHS with influent wastewater.
Table S14. Average removal and production rates of different nitrogen species in the PTS reactors.
Table S15. Metabolism of NOB in the three PTS reactors.
References (*46–93*)

Supplementary text

REPURE concept, example and configuration

Building on the review of current and leading-edge treatment and recovery technologies for wastewater resources (*15*, *25*, *29*, *30*, *34*, *46-52*), a conceptual model (REPURE version 1.0) was developed and analysed to assess emissions, products, and environmental effects attributable to repurposing wastewater elements (C, N, and P). Here, we considered wastewater collected from households only, without commercial or industrial wastewater, partly because municipal wastewater is abundant enough to consider its potential for resource recovery. For example, nearly 120 million cubic metres of municipal wastewater is generated daily in the US (*53*). We chose a design life of 50 years, typical for wastewater facilities. The model example was designed to address three challenges: (i) significantly reduce external inputs, including energy and chemicals, (ii) significantly curb unintended effects on the environment, and (iii) maximally harvest energy and materials from wastewater. Typical kinetic factor values for microbial communities used in the model are based on the above-mentioned literature, along with default values employed in the BioWin code. Wastewater characteristics, environmental conditions, and key design parameters, among other assumptions, are listed in table S1 and table S2, respectively. Even though environmental effects attributable to the construction and demolition phases of the system are substantially lower than are those of the operation phase (*54*), the corresponding construction inventory data for the REPURE process configuration are still provided for comprehensive analysis in future research efforts (see table S3).

Energy and substance balance

The demand side of the accumulative energy balance consists of the energy required for operational processes, such as aeration, mixing, and heating in reactors, liquid and sludge pumping, as well as sludge thickening and dewatering. The BioWin model was used to estimate the energy requirements, and the assumptions and methods are similar to those of our previous study (*4*). On the supply side, we assumed co-combustion of the obtained CH_4 with either the collected N_2O or external O_2 as oxidants, where 35% of the combustion enthalpy is harvested as electricity and 50% as heat in a combined heat and power system (*55*), both of which were represented in kWh. In addition, dewatered digested biosolids were assumed to be processed in a reformer for syngas production via incineration. Syngas production was calculated as a function of the dry solids content with the thermochemical equilibrium solver Cantera, and the energy conversion rates for electricity and heat were assumed to be 40% and 50%, respectively, according to the literature (*15*). A fraction of the recovered heat was used to heat the sludge for anaerobic digestion, as well as to partially or completely dry the solids. As regards the substance balance of the REPURE system, the total output of C, N, and P in the treated effluent, stabilised biosolids, and products (struvite and PHA), gases emitted (N_2, H_2, and CO_2), and energy carriers harvested (N_2O and CH_4), equalled the total input, C, N, and P in the incoming wastewater (influent), based on the integrative data sets obtained from the BioWin simulations.

Environmental emissions

Carbon emissions from the foreground processes were estimated from energy consumption, on-site organic matter degradation, and carbon release avoided through the generation of bioenergy from biogas combustion and the incineration of biosolids. As the generated CH_4 and N_2O were both considered as energy sources for recycling, the corresponding carbon footprint could be ignored. However, a CO_2 emission factor

for bioenergy conversion was still included. In particular, CO_2 conversion factors from coal-based energy production and biogas production were 877 and 353 g CO_2 kWh^{-1} respectively, whereas the CO_2 emission parameter for sludge incineration was 0.415 kg CO_2 kg^{-1} sludge (*26*). The accumulated carbon footprint is shown in fig. S4.

The net effect of each environmental category consisted of contributions from energy use in the system operation, transport of materials (inputs and products), avoided resources (manufacture, transport, and losses from application), release of organic and metallic pollutants, and emissions of ammonia, methane, and nitrous oxide, among others. Gaseous emissions from applications of effluent, biosolids, and struvite to the land were assumed and their amounts are provided in table S4. Additionally, the metal concentrations in the biosolids, struvite, and effluent were sourced from historical references, which are shown in table S5, table S6, and table S7, respectively. The concentrations of organic contaminants in the harvested products were sourced from previous studies, the organics in biosolids are presented in table S8, whereas the organics in effluent are presented in table S9. The assumed availability of nutrients in recovered fertilisers is presented in table S10. Transport of the captured products was also included. The transport inventories consisted of the total distance travelled, fraction of trip travelled with load (backhaul ratio), and the fraction of the trip in rural or urban areas (rural fractions), which were assumed in this work for the sake of easy analysis (table S11).

Ecosystem Service Benefits

To establish and explore the potential synergy between the technological and ecological components of the system, two key ecosystem services provided by soils were included. Carbon sequestration mediated by the soil system (median: 0.24 kg C kg^{-1} applied C; min: 0.05 kg C kg^{-1} applied C; max: 0.50 kg C kg^{-1} applied C) was sourced from the literature (*56*) and included at the systems level, while applying biosolids in land use practices. The soil system was also assumed to provide the benefits of nutrient retention, with only a minority of nutrients lost from biosolids (6.0% of applied N; 5.0% of applied P), struvite (6.7% of applied N; 5.3% of applied P), and irrigation (2.0% of applied effluent) to waterways (*19*, *57*).

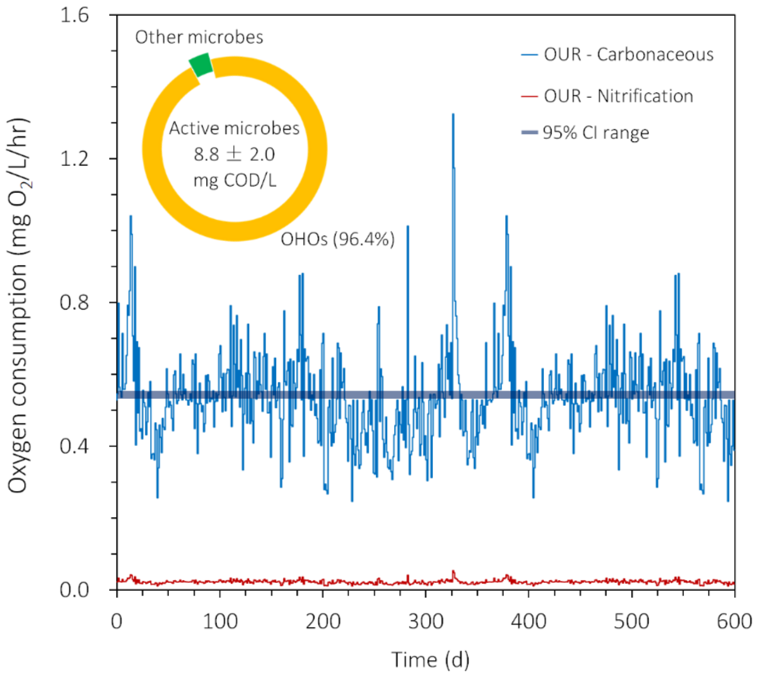

Fig. S1. Profiles of oxygen consumption and active microbes in CRR.

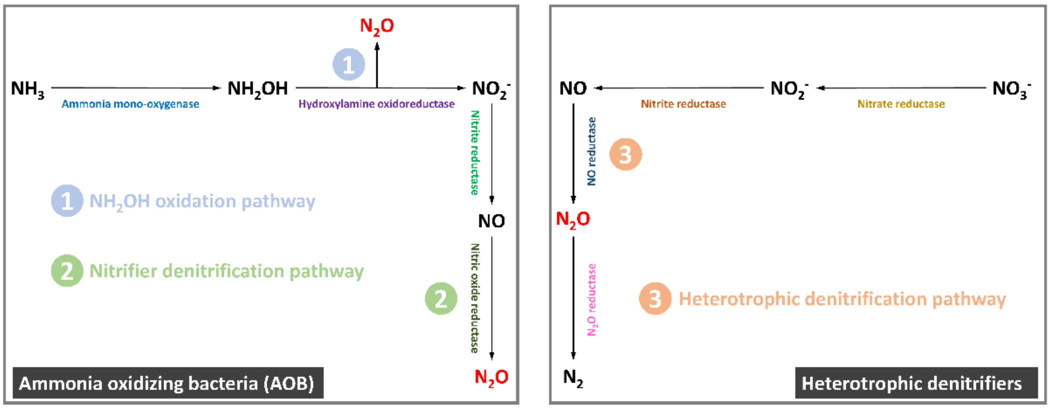

Fig. S2. Simplified illustration of the three key N_2O production pathways by AOB and heterotrophic denitrifiers. This illustration is based on the literature (*58*, *59*).

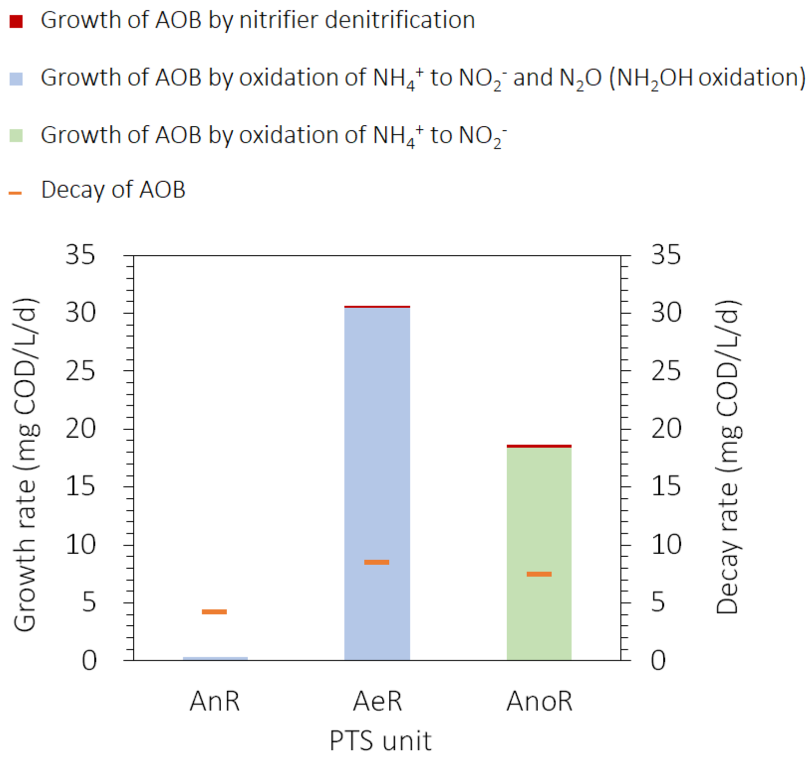

Fig. S3. Growth and decay rates of AOB in the PTS reactors.

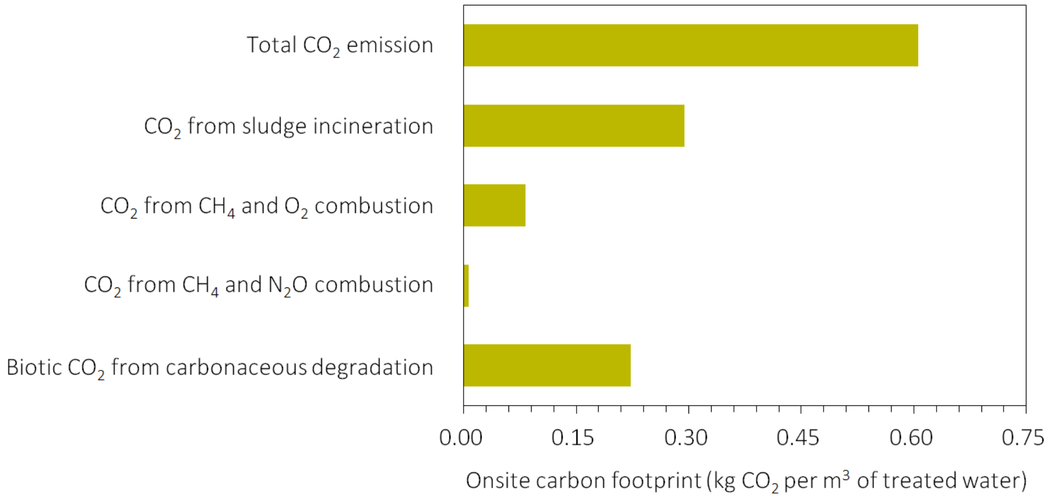

Fig. S4. Carbon footprint during operation of the REPURE process configuration.

Table S1. Environmental parameters and main characteristics of influent for process design and modeling. The data were captured and summarised based on a real-life data set of 600-day monitoring for a WWTP in northern China. Additional environmental parameters and influent characteristics used in the simulator were set as default values, except for those mentioned above.

Item	Unit	Median	Min	Max
Environmental parameters				
Flow rate	10^3 m^3	10	4.5	18
Ambient water and air temperature	°C	20	10	25
Plant altitude	m	40	-	-
Influent main characteristics				
pH	-	7.3	-	-
Alkalinity	mmol L^{-1}	6	-	-
Chemical oxygen demand (COD)	mg COD L^{-1}	400	201	957
Total Kjeldahl nitrogen (TKN)	mg N L^{-1}	40	20	60
Total phosphorus (TP)	mg P L^{-1}	7.0	2.5	9.8
Inorganic suspended solids (ISS)	mg ISS L^{-1}	25	-	-
Calcium	mg L^{-1}	80	-	-
Magnesium	mg L^{-1}	15	-	-

Table S2. Design parameters for the developed technological configuration.

Design parameter	Value
Substrate repurposing system (SRS)	
Hydraulic retention time	0.5 h for CRR; 1.2 d for CCR
Dissolved oxygen	2.0 mg O_2/L for CRR
Surface overflow rate	17 m^3 m^{-2} d^{-1} for 1st clarifier; 16 m^3 m^{-2} d^{-1} for 2nd clarifier
Return activated sludge ratio	79% for CRR; 0.14% for CCR
Operational temperature	20 °C
Partial treatment system (PTS)	
Solid retention time	3.5 d
Hydraulic retention time	0.6 h for AnaR; 1.2 h for AeR; 0.3 h for AnoR
Dissolved oxygen	2.0 mg O_2 L^{-1} for AeR; 0.5 mg O_2 L^{-1} for AnoR
Mixed liquid return ratio	120%
Surface overflow rate	20 m^3 m^{-2} d^{-1} for 3rd clarifier
Operational temperature	20 °C
Resources harvesting system (RHS)	
Hydraulic retention time	30 d for WRR
Alkalinity solution	30 kmol d^{-1}
Operational temperature	35 °C
Solids capture	95% for both thickening and dewatering units
Percent of contained water	95% after thickening; 85% after dewatering

Table S3. Construction inventory data for the REPURE process configuration. Basic calculation factors were derived from the literature to calculate the above inventory data (*60*).

Categories	Unit	Value
Construction inputs for major vessels		
Materials/assemblies		
Steel, Bluescope Port Kembla	t	4 446
Water consumption	t	6 991
Aluminium	t	50
Limestone	t	1 229
Chromium steel (stainless steel), 18% Cr, 8% Ni	t	357
Glass fibre, PA6 30%	t	112
Copper	t	53
EPDM (synthetic) rubber	t	50
Rock wool, mineral wool (insulation material)	t	50
Organic chemicals (use LDPE as proxy)	t	232
Bitumen	t	29
Inorganic chemicals (use phosphoric acid as proxy)	t	29
Low-density polyethylene (LDPE)	t	1
High-density polyethylene (HDPE)	t	140
Processes		
Excavation, hydraulic digger	t km	198
Transport, articulated truck 28/30 (freight)	t km	4 687 051
Transport, rail (bulk transport)	t km	5 543 824
Electricity consumption	kWh	2,293
Extrusion of PET	t	41
Construction inputs for aeration diffusers		
Materials/assemblies		
EPDM (synthetic) rubber	t	6
Polypropylene	t	12
Processes		
Transport, articulated truck 28/30 (urban)	t km	852
Construction inputs for pumps		
Materials/assemblies		
Mass of electronic motors	t	5
Mass of pumps, mixers, compressors, etc.	t	3
Processes		
Transport, articulated truck 28/30 (urban)	t km	392

.

Table S4. Default assumptions for gaseous emissions and attendant variability for uncertainty analysis. Triangle distribution was used to estimate the relevant uncertainties

Parameter	Unit	Median	Min	Max	References
Methane					
Biosolids in agriculture	kg CH_4 kg^{-1} DS	2.80×10^{-3}	0	9.60×10^{-3}	(*61*)
Nitrous oxide					
Biosolids in agriculture	kg N_2O kg^{-1} N applied	1.57×10^{-2}	4.71×10^{-3}	4.71×10^{-2}	(*60*)
Struvite application	kg N_2O kg^{-1} N applied	1.57×10^{-2}	4.71×10^{-3}	4.71×10^{-2}	(*60*)
Effluent application	kg N_2O kg^{-1} N applied	8.00×10^{-3}	2.00×10^{-3}	2.20×10^{-2}	(*61*)
Ammonia					
Biosolids in agriculture	kg NH_3 kg^{-1} N load	2.43×10^{-1}	6.08×10^{-2}	6.08×10^{-1}	(*60*)
Struvite application	kg NH_3 kg^{-1} N load	1.21×10^{-1}	3.64×10^{-2}	3.64×10^{-1}	(*60*)
Effluent application	kg NH_3 kg^{-1} N load	2.85×10^{-1}	1.82×10^{-1}	6.07×10^{-1}	(*62*)

Table S5. Heavy metal contaminants in biosolids. Triangle distribution was used to estimate the relevant uncertainties. The unit of the data in the table is mg kg^{-1}.

Element	Median	Min	Max	References
Arsenic	2.7	4.5	9.4	(*60*)
Cadmium	1.1	2.0	2.4	(*60*)
Chromium	10.8	23.2	39.2	(*60*)
Copper	201.2	280.0	459.4	(*60*)
Lead	12.2	37.0	62.0	(*60*)
Nickel	9.3	16.4	21.7	(*60*)
Zinc	212.7	492.8	775.5	(*60*)
Mercury	0.4	1.3	3.6	(*60*)
Selenium	2.9	3.7	5.5	(*60*)
Molybdenum	3.4	6.8	7.4	(*60*)

Table S6. Metal contaminants in struvite. Triangle distribution was used to estimate the relevant uncertainties. The unit of the data in the table is mg kg^{-1}.

Element	Median	Min	Max	References
Aluminium	96	11	588	(*63*, *64*)
Arsenic	7.5	0.48	15	(*64*, *65*)
Boron	4	1.8	29	(*64*, *66*)
Barium	2	0.5	9.7	(*64*)
Calcium	12100	1170	58000	(*63*, *64*)
Cadmium	0.1	0.06	4	(*64-68*)
Cobalt	0.15	0.15	1.7	(*64*)
Chromium	0.1	0.1	7.94	(*66*, *67*)
Copper	79.37	3	528	(*66*, *67*)
Iron	300	14	1050	(*63*, *66*, *69*)
Mercury	1.18	0.05	15	(*64*, *66-68*, *70*)
Lithium	0.5	0.5	2.1	(*64*)
Manganese	4.1	0.05	92.3	(*66*)
Molybdenum	0.5	0.5	3	(*64*)
Nickel	1.5	0.4	50	(*65-67*)
Lead	2.5	0.45	15.87	(*65-68*)
Sulfur	1350	400	3500	(*64*, *65*)
Selenium	3.7	2.9	5.5	(*66*)
Silicon	62.5	36	510	(*64*)
Tin	0.7	0.7	1.4	(*64*)
Strontium	22	4	45	(*64*)
Vanadium	0.05	0.05	1	(*64*)
Zinc	36	5	555.6	(*65-67*)

Table S7. Heavy metal concentrations in treated effluent. Triangle distribution was used to estimate the relevant uncertainties. "N/A" means no literature references were found. The unit of the data is μg L^{-1}.

Element	Median	Min	Max	References
Arsenic	0	0	0.85	(*71*)
Boron	235.8	0.05	1201.1	(*71*)
Calcium	79490	N/A	N/A	(*72*)
Cadmium	0.1	0	1.65	(*71*)
Cobalt	0.1	0.03	1.5	(*71*)
Chromium	0.35	0.15	2.7	(*71*)
Copper	0.05	0	1.3	(*71*)
Mercury	0	0	0.1	(*71*, *72*)
Iron	6.45	0.55	84.35	(*71*)
Nickel	0.05	0	0.85	(*71*)
Lead	0	0	0.8	(*71*)
Tin	0.05	0.05	17.5	(*71*)
Zinc	2.15	1	3159	(*71*)

Table S8. Organic contaminants in biosolids. Triangle distribution was used to estimate the relevant uncertainties

Compound	Unit	Median	Min	Max	References
1,2,3,6,7,8-Hexachlorodibenzo-p-dioxin	pg kg^{-1} DS	42.1	0.4	820	(*73*, *74*)
1,2,3,7,8-Pentachlorodibenzo-p-dioxin	pg kg^{-1} DS	3.5	1.3	15	(*73*, *74*)
2,3,4,5-Tetrachlorophenol	mg kg^{-1} DS	0.015	0.01	0.03	(15)
2,5-Dichlorophenol	µg kg^{-1} DS	0.056	0.01	0.15	(15)
2-Nitrophenol	mg kg^{-1} DS	0.09	0.025	0.5	(15)
3,4,5-Trichlorophenol	µg kg^{-1} DS	0.021	0.01	0.09	(15)
5-Chloro-2-(2,4-dichlorophenoxy) phenol	mg kg^{-1} DS	3.88	0.09	133	(*75-77*)
Acenaphthene	mg kg^{-1} DS	0.48	0.2	1.7	(15)
Aldrin	mg kg^{-1} DS	0.02	0.01	0.07	(*78*)
Anthracene	µg kg^{-1} DS	0.06	0.005	3	(15)
Benzo(a)anthracene	µg kg^{-1} DS	0.48	0.005	1.6	(15)
Benzo(a)pyrene	µg kg^{-1} DS	0.31	0.005	1.1	(15)
Bisphenol A	mg kg^{-1} DS	0.15	0	325	(*75*, *76*, *79*)
Chlordane	mg kg^{-1} DS	0.02	0.01	0.3	(*78*)
DDD	mg kg^{-1} DS	0.02	0.00114	84.1	(*78*, *80*)
DDE	mg kg^{-1} DS	0.02	0.01	0.27	(*78*)
DDT	mg kg^{-1} DS	0.04	0.01	3	(*78*, *81*)
Decabromodiphenyl oxide	mg kg^{-1} DS	0.46	0.0005	34.9	(*69*, *75*, *82-84*)
Dibenz(a,h)anthracene	µg kg^{-1} DS	0.16	0.1	2	(15)
Dibenzofuran, 2,3,7,8-tetrachloro-	µg kg^{-1} DS	8	0.8	52	(*73*, *74*)
Dieldrin	mg kg^{-1} DS	0.04	0.01	0.77	(*78*)
Dioxin,1,2,3,7,8,9-hexachlorodibenzo-	pg kg^{-1} DS	10.8	0.605	130	(*73*, *74*)
Dioxin,2,3,7,8 Tetrachlorodibenzo-p-	µg/kg DS	0.805	0.3	3.9	(*73*, *74*)
Erythromycin	mg kg^{-1} DS	0.04	0.002	0.18	(*75*)
Oestradiol	mg kg^{-1} DS	0.02	0.005	0.36	(*75*)
Estrone	mg kg^{-1} DS	0.016	0.001	0.965	(*75*)
Ethene, tetrachloro-	µg kg^{-1} DS	0.018	0.005	0.1	(15)
Ethinyl oestradiol	mg kg^{-1} DS	0.005	0.002	0.355	(*75*)
Fluoranthene	µg kg^{-1} DS	1.6	0.02	4	(15)
Fluorene	µg kg^{-1} DS	0.45	0.005	1.6	(15)
Heptachlor	mg kg^{-1} DS	0.03	0.02	0.17	(*78*)
Lindane	mg kg^{-1} DS	0	0	0.01	(*78*)
m-Cresol	mg kg^{-1} DS	0.08	0.025	0.46	(15)
Metacresol, parachloro-	µg kg^{-1} DS	0.019	0.01	0.06	(15)
Methane, dichloro-, HCC-30	mg kg^{-1} DS	0.525	0.025	1	(15)
m-Xylene	µg kg^{-1} DS	0.16	0.025	1.2	(15)
Naphthalene	mg kg^{-1} DS	0.71	0.005	3.2	(15)
o-Cresol	µg kg^{-1} DS	0.061	0.025	0.07	(15)
o-Xylene	µg kg^{-1} DS	0.093	0.05	0.7	(15)
P-(1,1,3,3-tetramethylbutyl) phenol	mg kg^{-1} DS	1.6	0.8	43.9	(*76*, *79*)
PAH, polycyclic aromatic hydrocarbons	mg kg^{-1} DS	6.3	3.38	9.15	(*85*)

p-Cresol	µg kg^{-1} DS	140	1.3	940	(15)
Phenanthrene	mg kg^{-1} DS	2	0.005	5.3	(15)
Phenol	mg kg^{-1} DS	12	0.1	43	(15)
Phenol, 2,3,4,6-tetrachloro-	mg kg^{-1} DS	0.016	0.01	0.04	(15)
Phenol, 2,4-dichloro-	µg kg^{-1} DS	0.056	0.01	0.15	(15)
Phenol, 2,4-dimethyl-	mg kg^{-1} DS	0.6	0.025	1.3	(15)
Phenol, 4-nitro-	mg kg^{-1} DS	0.14	0.025	0.7	(15)
Phenol, pentachloro-	mg kg^{-1} DS	0.02135	0.0071	0.239	(*73*, *79*)
Phthalate, butyl-benzyl-	µg kg^{-1} DS	0.38	0.01	1.3	(15)
Phthalate, dibutyl-	µg kg^{-1} DS	0.15	0.01	0.46	(15)
Phthalate, diethyl-	µg kg^{-1} DS	0.15	0.01	0.59	(15)
Phthalate, dimethyl-	µg kg^{-1} DS	0.13	0.01	1.5	(15)
Phthalate, dioctyl-	mg kg^{-1} DS	65	0.01	3514	(*75*, *85*)
P-nonylphenol	mg kg^{-1} DS	79	4.6	1230	(*76*, *79*, *85*)
Polychlorinated dioxins and furans	ng kg^{-1} DS	462.5	162	1036	(16)
p-Xylene	µg kg^{-1} DS	0.16	0.025	1.2	(15)
Pyrene	mg kg^{-1} DS	1.7	0.04	3.7	(15)
Tetracycline	mg kg^{-1} DS	1.28	0.04	5.27	(*75*)
Toluene	mg kg^{-1} DS	0.39	0.025	1.8	(15)
Triclocarban	mg kg^{-1} DS	19.3	0.19	441	(*75*)
Xylene	µg kg^{-1} DS	0.25	0.05	1.9	(15)

Table S9. Organic contaminants in treated effluent. Triangle distribution was used to estimate the relevant uncertainties. "N/A" means no literature references were found. The unit of the data in the table is μg L^{-1}.

Compound	Median	Min	Max	References
1,3-Dioxalane	1.7	N/A	N/A	(*86*)
1,8-Cineole	0.1	N/A	N/A	(*86*)
1-Methylnapthalene	2.15	0	4.3	(*86*)
2,3,4,5-Tetrachlorophenol	0.01	0	0.02	(*86*)
2,3,4-Trichlorophenol	0.01	0	0.02	(*86*)
2,3,5-Trichlorophenol	0.01	0	0.02	(*86*)
2,3,6-Trichlorophenol	0.01	0	0.02	(*86*)
2,3-Dichlorophenol	0.01	0	0.02	(*86*)
2,5-Dichlorophenol	0.06	0.06	0.13	(*86*)
2,6-Dichlorophenol	0.01	0	0.02	(*86*)
2-Hexanol	0.3	N/A	N/A	(*86*)
2-Hexanone	0.6	N/A	N/A	(*86*)
2-Phenoxyethanol	24.8	N/A	N/A	(*86*)
3,4,5-Trichlorophenol	0.01	0	0.02	(*86*)
3,4-Dichlorophenol	0.01	0	0.02	(*86*)
3,5-Dichlorophenol	0.01	0	0.02	(*86*)
3-Hexanol	0.7	N/A	N/A	(*86*)
5-Chloro-2-(2,4-dichlorophenoxy) phenol	0.4	0.075	5.9	(*86-88*)
5-Hepten-2-one, 6-methyl-	0.1	N/A	N/A	(*86*)
Acenaphthene	0.05	0.02	0.26	(*87*, *88*)
Acetamide	8.6	N/A	N/A	(*86*)
Acetaminophen	1.5	N/A	N/A	(*86*)
Adipate, bis(2-ethylhexyl)-	1	N/A	N/A	(*86*)
Anthracene	0.02	0.01	0.04	(*87*, *88*)
Benzene	0.95	0	1.9	(*86*)
Benzene, 1,2,4-trimethyl-	4.75	0	9.5	(*86*)
Benzene, 1,3,5-trimethyl-	4.8	0	9.6	(*86*)
Benzene, 1-methyl-2-nitro-	1.8	0	3.6	(*86*)
Benzene, ethyl-	2	1.9	2.1	(*86*)
Benzo(a)anthracene	0.005	0	0.01	(*87*, *88*)
Benzo(a)pyrene	0.0175	0.005	0.04	(*87*, *88*)
Benzoic acid	0.5	N/A	N/A	(*86*)
Butylated hydroxyanisole	0.5	N/A	N/A	(*86*)
Butyric acid, butyl ester	0.9	N/A	N/A	(*86*)
Caffeine	0.5	N/A	N/A	(*86*)
Coumarin	1	N/A	N/A	(*86*)
Cumene	2.75	0	5.5	(*86*)
Cyclododecane	8.1	N/A	N/A	(*86*)
Decabromodiphenyl oxide	0.005	0	0.01	(*87*, *88*)
Decane	4.2	N/A	N/A	(*86*)

Decanoic acid	380.25	5.5	755	(*86*)
Dibenz(a,h)anthracene	0.01	0	0.03	(*87*, *88*)
Di-isobutylphthalate	5.95	0.5	8	(*86-88*)
Dipropyl phthalate	0.5	0	1	(*86-88*)
Dodecanoic acid	342.95	5.9	680	(*86*)
Dodecanol	11.3	N/A	N/A	(*86*)
Eugenol	1	N/A	N/A	(*86*)
Fluoranthene	0.03	0.03	0.04	(*87*, *88*)
Fluorene	0.0535	0.0048	0.1	(*87*, *88*)
Geraniol	0.8	N/A	N/A	(*86*)
Hexanoic acid	6.05	0.3	11.8	(*86*)
Hexanol, 2-ethyl-1-	8.5	N/A	N/A	(*86*)
Indole	3.8	N/A	N/A	(*86*)
Malathion	1.9	N/A	N/A	(*86*)
m-Xylene	3.5	3.4	3.6	(*86*)
N,n-dimethyldodecylamine	7.4	N/A	N/A	(*86*)
Naphthalene	0.05	0.029	4.5	(*86-88*)
Naphthalene, 2-methyl-	2.25	0	4.5	(*86*)
Nicotine	1.2	N/A	N/A	(*86*)
Nitrobenzene	7.5	0	15	(*86*)
N-propylbenzene	2.8	0	5.6	(*86*)
Octanoic acid	145.5	8.1	283	(*86*)
o-Xylene	0.6	0.5	0.7	(*86*)
P-(1,1,3,3-tetramethylbutyl) phenol	0.11	0.07	0.2	(*86-88*)
p-Cresol	3.1	N/A	N/A	(*86*)
Phenanthrene	0.0625	0.015	0.12	(*87*, *88*)
Phenol,2,3,4,6-tetrachloro-	0.01	0	0.02	(*86*)
Phenol,2,4,5-trichloro-	0.01	0	0.02	(*86*)
Phenol,2,4,6-trichloro-	0.02	0	0.1	(*86*)
Phenol,2,4-dichloro-	0.06	0.06	0.13	(*86*)
Phenol,2,6-bis(1,1-dimethylethyl)-4-methyl-	4.5	N/A	N/A	(*86*)
Phenol, pentachloro-	0.025	0.01	0.05	(*86*, *89*)
Phosphate, tris(2-chloroethyl)-	0.4	N/A	N/A	(*86*)
Phthalate, butyl-benzyl-	2.5	0.5	9	(*86-88*)
Phthalate, dibutyl-	5.2	0.5	9.4	(*86-88*)
Phthalate, diethyl-	14.75	0.5	38	(*86-88*)
Phthalate, dimethyl-	0.5	0	1	(*86-88*)
Phthalate, dioctyl-	21.75	8.4	160	(*86-89*)
Phthalate, n-dioctyl-	0.375	0	1	(*87*, *88*)
P-hydroxybenzoic acid	1	N/A	N/A	(*86*)
P-nonylphenol	0.76	0.4	5.96	(*86-89*)
p-Xylene	3.5	3.4	3.6	(*86*)
Pyrene	0.03	0.01	0.05	(*87*, *88*)
Salicylic acid	0.6	N/A	N/A	(*86*)
Sulfuric acid, dimethyl ester	0.1	N/A	N/A	(*86*)

Tetrabutyltin	0.0005	0	0.001	(*86*)
Thymol	2.5	N/A	N/A	(*86*)
Toluene	1.4	N/A	N/A	(*86*)
Triphenylphosphate	0.5	N/A	N/A	(*86*)

Table S10. Assumed availability of nutrients in recovered fertilizers as a fraction of commercial fertilizer availability. Triangle distribution was used to estimate the relevant uncertainties.

Product	Nitrogen		Phosphorus		References
	Value	Uncertainty[a]	Value	Uncertainty[a]	
Biosolids	50	25–85	70	25–100	(*54*, *90*-*92*)
Struvite	100	-	100	-	(*93*)

Table S11. Transport assumption and distances for recovered and commercial fertilizers. The assumptions and data used in this table were referred from the literature (*41*). Triangle distribution was used to estimate the relevant uncertainties. The backhaul ratio represents the distance travelled with load. A ratio of 2 means the truck is fully loaded there and back, whereas a value of 1 means the truck is empty on the return journey.

	Backhaul ratio[b]	Rural fraction	Distance (km)	Distance range for uncertainty	Rationale
Biosolids to agriculture	1	0.75	200	25-600	Rural use from urban center, restricted application
Struvite	1	0.60	50	25-600	Rural use from urban center, closer application than biosolids

Table S12. Removal efficiencies of effluent COD, TN, and TP for the REPURE system.

	Mean	SD	Median	95% Confidence interval		Percentiles of the distribution			
				Lower	Upper	5%	25%	75%	95%
COD	92	4	93	92	93	90	92	94	95
TN	81	4	81	80	81	76	79	83	85
TP	93	4	93	93	93	89	92	94	95

Table S13. Comparison of the average concentration of the major carbon substances in the outflow from CRR, CCR, and RHS with influent wastewater.

Concentration	Influent	CRR outflow stream	CCR outflow stream	RHS fermentation liquid
Readily biodegradable COD (mg L^{-1})	50	64	127	104
HAc (mg COD L^{-1})	8.3	9.6	115	102

Table S14. Average removal and production rates of different nitrogen species in the PTS reactors. The unit of the data is mg N L^{-1} h^{-1}.

PTS units	Nitrification				Denitrification			
	NH_4^+ removal	NO_2^- production	NO_3^- production	N_2O production	NO_3^- removal	NO_2^- removal	N_2O production	N_2 production
AnR	0.05	0.02	0	0.08	0.26	3.8	0.04	4.0
AeR	7.2	2.7	0.21	4.4	0.03	0.05	0	0.08
AnoR	4.6	2.5	0.13	2.1	0.09	0.13	0.01	0.21

Table S15. Metabolism of NOB in the three PTS reactors.

	NOB metabolic profile	
	Average growth (mg COD L^{-1} d^{-1})	Average decay (mg COD L^{-1} d^{-1})
AnR	0	0.11
AeR	0.71	0.21
AnoR	0.44	0.18
